# Susceptibility of common family *Anatidae* bird species to clade 2.3.4.4e H5N6 high pathogenicity avian influenza virus: an experimental infection study

**DOI:** 10.1186/s12917-022-03222-7

**Published:** 2022-04-02

**Authors:** Kosuke Soda, Yukiko Tomioka, Chiharu Hidaka, Mayu Matsushita, Tatsufumi Usui, Tsuyoshi Yamaguchi

**Affiliations:** Faculty of Agriculture, Avian Zoonosis Research Center, Tottori University, Tottori, 680-8553 Japan

**Keywords:** Clade 2.3.4.4, Duck, H5N6, High pathogenicity avian influenza, Mallard, Pintail, Wigeon

## Abstract

**Background:**

There were large outbreaks of high pathogenicity avian influenza (HPAI) caused by clade 2.3.4.4e H5N6 viruses in the winter of 2016–2017 in Japan, which caused large numbers of deaths among several endangered bird species including cranes, raptors, and birds in Family *Anatidae*. In this study, susceptibility of common *Anatidae* to a clade 2.3.4.4e H5N6 HPAI virus was assessed to evaluate their potential to be a source of infection for other birds. Eurasian wigeons (*Mareca penelope*), mallards (*Anas platyrhynchos*), and Northern pintails (*Anas acuta*) were intranasally inoculated with 10^6^, 10^4^, or 10^2^ 50% egg infectious dose (EID_50_) of clade 2.3.4.4e A/teal/Tottori/1/2016 (H5N6).

**Results:**

All birds survived for 10 days without showing any clinical signs of infection. Most ducks inoculated with ≥ 10^4^ EID_50_ of virus seroconverted within 10 days post-inoculation (dpi). Virus was mainly shed via the oral route for a maximum of 10 days, followed by cloacal route in late phase of infection. Virus remained in the pancreas of some ducks at 10 dpi. Viremia was observed in some ducks euthanized at 3 dpi, and ≤ 10^6.3^ EID_50_ of virus was recovered from systemic tissues and swab samples including eyeballs and conjunctival swabs.

**Conclusions:**

These results indicate that the subject duck species have a potential to be a source of infection of clade 2.3.4.4e HPAI virus to the environment and other birds sharing their habitats. Captive ducks should be reared under isolated or separated circumstances during the HPAI epidemic season to prevent infection and further viral dissemination.

**Supplementary Information:**

The online version contains supplementary material available at 10.1186/s12917-022-03222-7.

## Background

To date, H5 subtype high pathogenicity avian influenza viruses (HPAIVs) have spread to domestic poultry and wild birds in over 70 countries in Asia, Europe, the Middle East, and Africa since late 1996 when the virus, recognized as the precursor of present circulating viruses (Goose/Guangdong [Gs/GD]-like viruses) was isolated from a goose in Guangdong Province, China [1]. Initial reports of high pathogenicity avian influenza (HPAI) were largely confined to poultry populations, and lethal Gs/GD-like virus infection was not detected in wild birds. In 2002, however, outbreaks of Gs/GD-like viruses were reported for the first time in waterfowl and other captive/wild birds in two waterfowl parks in Hong Kong [2]. Subsequently, more than a thousand migratory birds on Lake Qinghai in China died from HPAIV infection in May and June 2005 [3]. Multiple HPAI outbreaks in wild birds have since been reported at numerous locations around the world. Phylogenetically, based on the hemagglutinin (HA) genes, Gs/GD-like viruses have been organized into 10 clades, numbered clades 0–9 [4]. Among them, clade 2.3.4.4 HPAIVs have been selected as major circulating strains worldwide since late 2013 [5]. The clade 2.3.4.4 HPAIVs had genetically evolved and grouped into four clusters, A–D [6]. Subsequently, these viruses have been reclassified into eight clusters (a–h) by WHO [7]. In this article, the WHO classification was used to describe the subclades of clade 2.3.4.4 HPAIVs, even when citing publications that used the previous classification scheme.

In the winter of 2016–2017, clade 2.3.4.4e (previously, Group C) H5N6 HPAIVs caused many outbreaks of HPAI in poultry and wild birds in the East Asian countries [8–10]. Japan experienced the largest HPAI outbreaks in wild birds. A total of 218 wild/captive birds, including endangered species, died as a result of infection or were euthanized as a preventive measure [11, 12]. Successive outbreaks occurred in Izumi Plain in Kagoshima Prefecture, which is a major overwintering site for migratory birds [11]. Virus infection was confirmed in dead/debilitated hooded cranes (*Grus monachal*) and white-naped cranes (*Grus vipio*), which are classified as Vulnerable according to the International Union for Conservation of Nature and Natural Resources (IUCN) Red List of Threatened Species [13]. Reared cackling geese (*Branta hutchinsii leucopareia*, a Critically Endangered category in the IUCN Red List) in Higashiyama Zoo and Botanical Gardens in Aichi Prefecture also died of infection [12]. Viral infections were coincidentally observed in Family *Anatidae* bird species including Eurasian wigeons (*Mareca penelope*) and Northern pintails (*Anas acuta*) sharing their habitat with cranes in the Izumi Plain, and mallards (*Anas platyrhynchos*) and Eurasian wigeons co-housed with the cackling geese in Higashiyama Zoo. These incidents suggest that the risk of HPAIV infections in endangered bird species is correlated with the viral susceptibility of *Anatidae*.

Several groups have examined pathogenicity of clade 2.3.4.4c (Group A) H5N2 or H5N8 HPAIVs, which circulated worldwide in 2014–2015, in *Anatidae* such as the American black duck (*Anas rubripes*), Baikal teal (*Anas formosa*), common teal (*Anas crecca*), Eurasian wigeon, lesser scaup (*Aythya affinis*), mallard, Mandarin duck (*Aix galericulata*), pintail, ruddy duck (*Oxyura jamaicensis*), and surf scoter (*Melanitta perspicillata*) [14–23]. Most studies showed that clade 2.3.4.4c HPAIVs caused subclinical infection in *Anatidae*, and that the viruses were mainly shed via the oral route. Little is known about the pathogenicity of clade 2.3.4.4e HPAIVs in *Anatidae.* Mandarin ducks inoculated with the clade 2.3.4.4e HPAIVs showed inapparent infection with virus shedding via the oral route [24]; Wang et al. [25] reported that some 2.3.4.4e HPAIVs demonstrated relatively higher pathogenicity in mallards, accompanied by the excessive expression of iNOS in the brain.

In the present study, we assessed susceptibilities of common *Anatidae* (Eurasian wigeon, mallard, and Northern pintail) to a clade 2.3.4.4e H5N6 HPAIV to assess their importance as a source of infection to environment and other birds, including threatened species, and to determine why clade 2.3.4.4e HPAIVs caused HPAI across East Asian countries. Migratory flyways of these ducks in East Asia were tracked using global positioning system technology in addition to classical bird banding studies [26–29], and revealed that they were probably involved in transboundary HPAIV dissemination. The results of this study contribute to the understanding of the 2016–2017 outbreaks, and may help to respond better to future HPAI outbreaks.

## Results

Eight of 30 captured ducks (3 Eurasian wigeons, 4 mallards, and 1 Northern pintail) were seropositive for influenza A virus by competitive enzyme-linked immunosorbent assay (cELISA) (see Additional file 1). Among them, 2 wigeons and 1 mallard had low titers (2–4) of hemagglutination inhibition (HI) antibody against the challenge strain. Another mallard (M120) had an H7N7 subtype low pathogenicity avian influenza virus detected in a cloacal sample. Based on these results and the criteria as described in Materials and Method section, the ducks were grouped and applied for the subsequent infectious experiment (The experimental designs are described in Additional file 2).

Each of seven Eurasian wigeons, mallards, and Northern pintails was inoculated with 10^6–2^ 50% egg infectious dose (EID_50_) of the clade 2.3.4.4e HPAIV A/teal/Tottori/1/2016 (H5N6) (Tottori/1) and observed for 10 days (Table 1). All the ducks survived without showing any clinical signs during the observation period. The HI testing showed that all the ducks inoculated with 10^6^ EID_50_ of the virus (W104–W106, M114–M116, and P124–P126) seroconverted against the challenge virus by 10 days post inoculation (dpi). Among them, some ducks, including one cELISA-positive mallard (M115), shed viruses via oral route for a maximum of 10 days, peaked at 3–5 dpi. One each of wigeon and mallard, W106 and M116, respectively, also shed a relatively low titer of virus via the cloacal route. Virus was additionally recovered from a conjunctival swab of one mallard, M116, at 10 dpi, despite no virus shedding being detected in oral/cloacal samples at 7 dpi. Among the ducks inoculated with 10^4^ EID_50_ of the virus, one juvenile wigeon and one juvenile mallard, W108 and M117, respectively, seroconverted (128–256 HI titers) and shed virus via the oral and/or cloacal routes. Viral shedding and antibody responses were not observed in an adult wigeon and an adult mallard (W107 and M118, respectively) which had a pre-HI antibody titer of 2, or two Northern pintails (P127 and P128). None of the ducks inoculated with 10^2^ EID_50_ of the virus shed any virus. Among them, one wigeon, W109, showed 4-fold change in its serum HI titer after the virus challenge. Notably, throughout the experiment, relatively low titers of viruses remained in the pancreas of several ducks at 10 dpi. No histopathological lesions or antigen-positive cells were observed in the tissues of ducks euthanized at 10 dpi (data not shown).

**Table 1:** Antibody responses and viral titers in the swabs, bloods, and tissues of *Anatidae* intranasally inoculated with clade 2.3.4.4 H5N6 HPAIV and observed for 10 days

Species	Inoculum titer (log EID_50_)	ID	Sex^a^	Age^b^	Serum antibody			Viral titer (log EID_50_/mL)									Viral titer (log EID_50_/g)	
					cELISA	HI titer		Pharyngolaryngeal / cloacal swabs							Conjunctival swab	Blood	Tissues	
					Pre	Pre	Post	0d	1d	2d	3d	5d	7d	10d			Pancreas	Other tissues^c^
Eurasian wigeon	6	W104	M	J	+	< 2	32	− ^d^/ −	− / −	− / −	− / −	− / −	− / −	− / −	−	−	−	−
		W105	M	A	−	< 2	32	− / −	− / −	− / −	− / −	− / −	− / −	− / −	−	−	−	−
		W106	M	J	−	< 2	32	− / −	≤ 1.0 / −	≤ 0.6 / −	4.3 / 2.5	2.7 / 2.5	− / −	− / −	−	−	≤ 1.8	−
	4	W107	M	A	+	2	4	− / −	− / −	− / −	− / −	− / −	− / −	− / −	−	−	−	−
		W108	M	J	−	< 2	128	− / −	4.5 / −	1.2 / −	4.0 / 3.5	4.5 / 2.5	− / 2.5	− / ≤ 1.2	−	−	−	−
	2	W109	M	A	+	4	16	− / −	− / −	− / −	− / −	− / −	− / −	− / −	−	−	−	−
		W110	M	J	−	< 2	< 2	− / −	− / −	− / −	− / −	− / −	− / −	− / −	−	−	−	−
Mallard	6	M114	M	J	−	< 2	64	− / −	3.5 / −	− / −	− / −	− / −	− / −	− / −	−	−	≤ 1.4	−
		M115	F	J	+	< 2	64	− / −	≤ 1.3 / −	1.5 / −	6.3 / −	2.5 / −	3.3 / −	≤ 0.4 / −	−	−	−	−
		M116	M	A	−	< 2	16	− / −	2.3 / −	1.7 / ≤ 1.3	4.5 / −	≤ 1.0 / −	− / −	− / −	2.5	−	≤ 1.4	−
	4	M117	F	J	−	< 2	256	− / −	4.2 / −	3.7 / −	3.7 / −	− / −	− / −	− / −	−	−	−	−
		M118	M	A	+	2	4	− / −	− / −	− / −	− / −	− / −	− / −	− / −	−	−	−	−
	2	M119	F	J	−	< 2	< 2	− / −	− / −	− / −	− / −	− / −	− / −	− / −	−	−	−	−
		M120	M	J	+	< 2	< 2	− / −	− / −	− / −	− / −	− / −	− / −	− / −	−	−	−	−
Northern pintail	6	P124	F	A	−	< 2	128	− / −	2.5 / −	1.7 / −	− / −	3.7 / −	≤ 0.6 / −	− / −	−	−	≤ 2.0	−
		P125	M	A	−	< 2	128	− / −	2.7 / −	3.5 / −	5.5 / −	− / −	− / −	− / −	−	−	−	−
		P126	F	J	−	< 2	64	− / −	2.2 / −	≤ 0.7 / −	≤ 0.7 / −	≤ 3.6 / −	− / −	− / −	−	−	−	−
	4	P127	F	J	−	< 2	< 2	− / −	− / −	− / −	− / −	− / −	− / −	− / −	−	−	−	−
		P128	F	A	−	< 2	< 2	− / −	− / −	− / −	− / −	− / −	− / −	− / −	−	−	−	−
	2	P129	F	A	−	< 2	< 2	− / −	− / −	− / −	− / −	− / −	− / −	− / −	−	−	≤ 1.6	−
		P130	F	J	−	< 2	< 2	− / −	− / −	− / −	− / −	− / −	− / −	− / −	−	−	≤ 1.3	−

^a^ *F* female, *M* male

^b^ *A* adult, *J* juvenile

^c^ including brain, trachea, breast muscle, lung, liver, spleen, heart, kidney, colon, eyeball, and wingshaft

^d^ indicates that the swab/blood and tissue tested negative for virus isolation (lower limit = 0.5 log EID_50_/g and 1.5 log EID_50_/g, respectively)

**Fig. 1:**
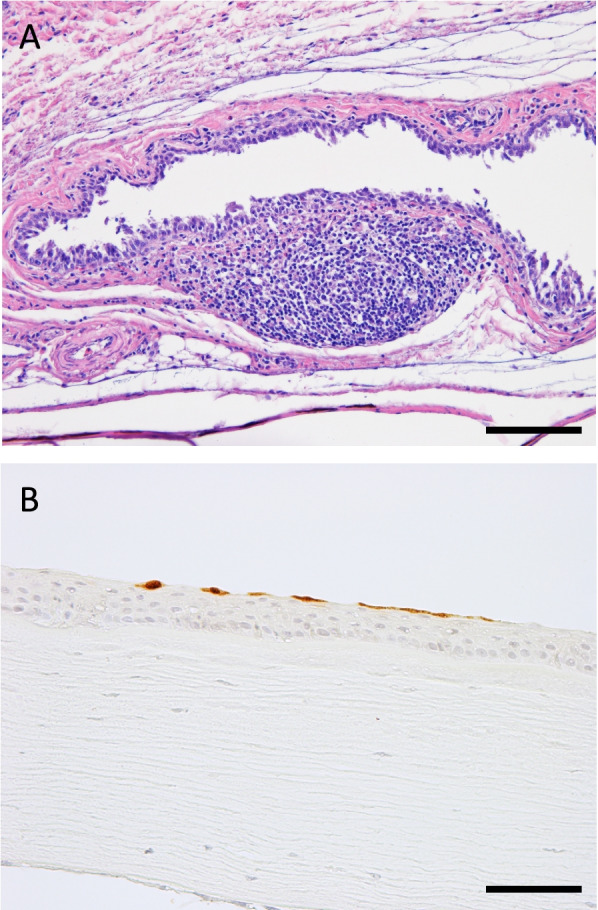
Conjunctivitis caused by clade 2.3.4.4e H5N6 high pathogenicity avian influenza virus in a Northern pintail duck. Representative histopathological findings of conjunctivitis (**A**) and immunohistochemical demonstration of type A influenza virus antigens in the corneal epithelium (**B**), The specimen is from a Northern pintail (P121), collected at 3 days post-infection. *Bars* indicate 100 µm (**A**) and 50 µm (**B**)

Viral tropism was assessed in the euthanized ducks at 3 dpi with 10^6^ EID_50_ of the virus (Table 2). None of the ducks showed any clinical signs during the observation period. Viruses at titers of ≤ 10^6.3^ EID_50_ were recovered from all the samples (other than the wing shafts) of a wigeon and a pintail (W102 and P121, respectively) and viremia was confirmed. Systemic infection was also observed in one pintail (P123) with viruses disseminated to various tissues at titers of ≤ 10^6.5^ EID_50_. Notably, the three ducks with systemic infection shed relatively higher titers of the virus (10^4.5–5.7^ EID_50_) via conjunctival route, accompanied by viral replication in their eyeballs. Three mallards (M111–M113) and one Northern pintail (P122) had relatively low titers of the virus in the limited tissues. No virus was isolated from two Eurasian wigeons (W101 and W103). None of the ducks had any obvious histopathological lesions at 3 dpi, other than the eyes of two pintails (P121 and P123), which showed mild nonsuppurative conjunctivitis which was composed of subconjuncitival inflammatory infiltrate and epithelial degeneration (Fig. 1A). Immunohistochemistry confirmed a small number of viral antigen-positive cells in some tissues of one wigeon (W102) and two pintails (P121 and P123), with the viruses at titers of ≥ 10^4^ EID_50_/g (Table 2 and Fig. 1B).

**Table 2:** Viral titers and antigen detection in the *Anatidae* intranasally inoculated with clade 2.3.4.4 H5N6 HPAIV^a^

Species	Eurasian wigeon			Mallard			Northern pintail		
ID	W101	W102	W103	M111	M112	M113	P121	P122	P123
Sex^b^	M	M	M	M	F	M	F	F	F
Age^c^	A	J	A	J	J	J	A	J	J
pre cELISA	−	−	−	−	+	−	−	−	−
pre HI titers	<2	<2	<2	<2	<2	<2	<2	<2	<2
Viral titers in tissues (log EID_50_/g)									
Brain	-^d^	2.5	−	−	−	−	4.3	−	−
Trachea	−	3.5	−	−	−	−	2.5	−	3.7
Breast muscle	−	3.5	−	−	−	−	≤2.0	−	−
Lung	−	5.3^e^	−	−	−	−	5.7^e^	2.5	2.7
Liver	−	4.7^e^	−	−	−	−	4.7^e^	−	−
Pancreas	−	3.3	−	−	−	−	≤2.0	2.5	4.7^e^
Spleen	−	4.3^e^	−	−	−	−	5.0^e^	−	4.0
Heart	−	4.0	−	−	4.5	≤1.7	4.0^e^	2.7	−
Kidney	−	4.7	−	2.5	−	−	4.5^e^	−	4.5
Colon	−	3.7	−	−	−	−	5.5^e^	≤1.4	6.5
Eyeball	−	4.7	−	−	−	−	6.3^e^	−	5.5^e^
Wing shaft	−	−	−	−	−	−	−	−	−
Viral titers in blood and swabs (log EID_50_/mL)									
Blood	−	4.5	−	−	−	≤0.7	3.5	−	−
Pharyngolaryngeal swab	−	3.5	−	−	−	−	5.5	2.3	4.7
Cloacal swab	−	≤0.6	−	−	−	−	≤1.0	−	−
Conjunctival swab	−	4.5	−	−	−	−	5.7	−	5.5

^a^ The samples were collected 3 days post-innoculation

^b^ *F* female, *M* male

^c^ *A* adult, *J* juvenile

^d^ indicates that the swab/blood and tissue tested negative for virus isolation (lower limit = 0.5 log EID_50_/g and 1.5 log EID_50_/g, respectively)

^e^ Viral antigen-positive cells were confirmed by immunohistochemistry

## Discussion

In this study, three adult *Anatidae* captured in the winter of 2018–2019 (W107, W109, and M118) had low titers of serum HI antibody against the challenge virus before the infection experiments, indicating that they were infected with antigenically similar influenza viruses and subsequently survived in the field. Other reports also showed that H5 HA specific antibody was detected in serum and egg yolk of wild duck populations in Asian countries [30, 31]. The HI seropositive *Anatidae* did not shed the virus after the challenge of the clade 2.3.4.4e H5N6 HPAIV. As observed in a previous study: mallards and Mandarin ducks with homologous immunity did not contribute to virus transmission [32, 33]. Antibody raised against HPAIV infection in wild ducks is likely to be maintained as observed in a surviving mallard in a zoo [12]. These results indicate that wild duck are involved in transboundary dissemination of HPAIV. In island countries such as Japan, increases and decreases in the seroprevalence in migratory ducks is probably correlated with the occurence of HPAI every few years [34]. The results of this study support this hypothesis as no ducks captured in 2019–2020 had pre-HI antibody against the challenge virus, in contrast to those captured in 2018–2019 (See Additional file 1); HPAI outbreaks subsequently occurred in Japan in the winter of 2020–2021 [35]. The challenge virus was recovered from two cELISA-positive juvenile mallards, suggesting that heterologous antibody is less likely to inhibit propagation of HPAIV in *Anatidae*.

Most of the *Anatidae* inoculated with ≥ 10^4^ EID_50_ of Tottori/1 showed subclinical infection. Similar results have been reported in Mandarin ducks with clade 2.3.4.4e H5N6 HPAIVs [24, 33]. In another study, most mallards died of infection within 8 weeks after being infected with clade 2.3.4.4e A/*Pavo cristatus*/Jiangxi/JA1/2016 (H5N6) [25], while those infected with another clade 2.3.4.4 strain, A/*Anas crecca*/shanghai/SH1/2016 (H5N6), caused no signs of infection. These results suggest that pathogenicity of clade 2.3.4.4e HPAIVs in *Anatidae* varies depending on the strain. Clade 2.3.4.4e H5N6 HPAIVs have multiple gene constellations (C1–C8 and more) [6, 9, 11, 36, 37]. Among them, the group C2 strains, including Tottori/1, were the most widely distributed strains in Japan in the winter of 2016–2017 [9]. The relatively low pathogenicity of the C2 strains probably contributes efficient virus transport by migratory *Anatidae* flocks. The ages of the duck subjects should also be considered to explain the differences in the pathogenesis of clade 2.3.4.4e H5N6 HPAIVs. Previous reports revealed that the past H5N1 HPAIVs showed higher pathogenicity in younger domestic ducks [38, 39]. Juvenile *Anatidae* captured in this study were presumed to be several months old and to have been born during the summer in Northern nesting areas such as Siberia and Alaska, and then migrated to Japan for wintering. The pathogenicity of Tottori/1 in younger ducks warrants further study.

Virus shedding from the infected *Anatidae* was mainly observed via the oral route as in previous experimental infection studies of *Anatidae* (Eurasian wigeons and mallards) and clade 2.3.4.4b and 2.3.4.4c H5 HPAIVs [14, 20]. Similarly, some ducks in this study orally shed the virus at titers of ≥ 10^4^ EID_50_, in 1–5 dpi, suggesting that these duck species might be a source of infection for other wild bird species in field settings. Further, two Eurasian wigeons shed virus into the cloaca in late phase of infection. A similar observation was made in Mandarin ducks infected with a clade 2.3.4.4c H5N8 HPAIV [16]. One possibility is that the viruses orally shed into drinking water were retaken, and entered the digestive tracts under the experimental conditions. Alternatively, virus that replicated in the respiratory tract may have spread hematogenously. Notably, several *Anatidae*, including two Northern pintails inoculated with 10^2^ EID_50_ of the virus, had low titers of virus in the pancreas at 10 dpi. Such tissue tropism was also observed in Baikal teals that died of natural infection caused by a clade 2.3.4.4c H5N8 HPAIV [40]. It remains unclear why the HPAIVs showed high tissue tropism for the pancreas in *Anatidae*. Pancreatic enzyme activity may contribute to cleavage activation of viral HA and isolation efficiency in experimental studies. In this study, virus detection in the pancreas and cloacal swabs was not correlated, indicating that the remaining viruses in the pancreas were not involved in virus shedding via the digestive tract.

Viremia and systemic infection were confirmed in two *Anatidae*. Their clinical outcomes after 3 dpi were unclear. Generally, such *Anatidae* are likely to be weakened and/or dead in field settings where birds have to feed by themselves, and they then become a source of infection by consumption by scavengers. Virus at titers of ≥ 10^4.5^ EID_50_ was also recovered from the eyeballs and conjunctival swabs of three ducks, and mild conjunctivitis were confirmed in the Northern pintails. Continuous virus detection in conjunctival swabs has been reported in domestic ducks inoculated with H5N1 HPAIVs [41], and antigen has been detected in ocular tissues [42]. Scavenging birds usually begin feeding on the eyes of carcasses, or attack live animals in certain instances, initially pecking out the eyes or feeding on open wounds [43]; HPAIVs in ocular tissues of ducks probably contribute to virus transmission.

## Conclusion

This study showed that ≥ 10^4^ EID_50_ of the clade 2.3.4.4e HPAIV caused inapparent infection in *Anatidae*, sometimes accompanied by viral shedding. The number of examined *Anatidae* was limited and their profiles (sex, age, and seroprevalence) were not uniform. However, the results reflect the circumstances of HPAIV infection in *Anatidae* populations in field settings. Age, history of infection, and individual differences may affect the clinical outcome. Transboundary dissemination of the virus by migratory birds, including *Anatidae*, is still a concern. Recently, clade 2.3.4.4b HPAIVs have become predominant in East Asian countries [44], and antigenically different clade 2.3.4.4h HPAIVs, evolved from clade 2.3.4.4e HPAIVs, have also been detected in China and Vietnam [45]. Assessing the pathogenicity using wild *Anatidae* should continue to be used to estimate the risk of infection in poultry and endangered species. The results of this study contribute to the accumulation of knowledge about the susceptibility of migratory ducks to H5 HPAIVs.

## Methods

### Virus

Clade 2.3.4.4e HPAIV Tottori/1 was used for experimental infection of ducks. The strain was isolated from a fecal sample of teal in Tottori City located in the Midwestern region of Japan [46]. The virus was categorized into the C2 group of the clade 2.3.4.4e, which has the most popular gene constellation in the 2016–2017 outbreak in Japan [9]. The accession numbers of the gene sequences were LC199865–199872. Virus was propagated in 10-day-old chicken embryos (Aoki Breeder Farm, Tochigi, Japan) for 48 h at 35°C. After the incubation period, eggs were chilled at 4°C for 12 h. The allantoic fluid was harvested and stored as virus stock at −80°C.

### Birds

Ten each of three *Anatidae* species (Eurasian wigeon, Mallard, and Northern pintail) were captured at Togo and Koyama Ponds in Tottori Prefecture in Japan in two consecutive winter seasons, January and December 2019 (see Additional file 1). Their ages (juvenile or adult) were identified by feather growth and molt. The pharyngolaryngeal, cloacal, and conjuntival swabs were collected and examined for influenza A virus antigen via rapid diagnostic kits (ESPLINE INFLUENZA A & B-N, Fujirebio Inc., Tokyo, Japan) and/or for virus isolation by egg inoculation. Blood was also collected, and subsequently checked for specific serum antibody against the challenge virus, Tottori/1, by HI testing [47]. These sera were also assayed for influenza A subtype viruses using a cELISA kit (IDEXX Influenza A Ab Test, IDEXX laboratories, ME, USA). The ducks were house at Tottori University for a maximum of 5 weeks. The ducks were grouped according to the following criteria: serum HI titers under the detection limit (< 2 HI) were preferentially applied to 10^6^ EID_50_ inoculation group as stated below; cELISA-positive ducks were impartially distributed to each group as possible; their age (adult/juvenile) and sex were also taken into consideration for grouping to reduce biases.

### Experimental design

Seven of each duck were intranasally inoculated with 200 μL of allantoic fluid containing the Tottori/1 at 10^6^, 10^4^, or 10^2^ EID_50_, then observed for clinical signs at 24-h intervals for 10 days (see Additional file 2). Pharyngolaryngeal and cloacal swabs were collected at 1, 2, 3, 5, 7, and 10 dpi to assess viral shedding. The swabs were collected in 2 mL of nutrient broth medium (Nissui Pharmaceutical, Tokyo, Japan) with 10 mg of streptomycin sulfate (Meiji Seika Pharma, Tokyo, Japan) and 1 × 10^4^ units of penicillin G (Meiji Seika Pharma). At the end of the 10-day period, the ducks were also checked for specific antibodies against the challenge virus in serum by HI testing. The surviving birds were euthanized by isoflurane (Fujifilm Wako Pure Chemical Corporation, Tokyo, Japan) inhalation at an overdose after collection of conjunctival swabs and blood at 10 dpi, and their tissues (brain, trachea, breast muscle, lung, liver, pancreas, spleen, heart, kidney, colon, eyeball, and wing shaft) were sampled for virus isolation and histopathological study, as described below. The remaining three of each duck species were intranasally inoculated with 200 μL of allantoic fluid containing the virus at 10^6^ EID_50_, then euthanized at 3 dpi. The samples were collected in the same manner as above.

Portions of the tissue samples were homogenized using a Multi-Bead Shocker (Micro Smash™ MS-100R, Tomy Seiko, Tokyo, Japan) at 3,000 rpm for 30 s to create a 10% (weight/volume) organ emulsion in nutrient broth medium with antibiotics. Samples serially tenfold diluted in phosphate buffered saline with streptomycin sulfate and penicillin G were inoculated into 10-day-old chicken embryos. Eggs were incubated at 35°C for 48 h. Hemagglutination testing [48] was then performed using allantoic fluid, and the EID_50_ was calculated using the Reed and Müench method [49]. The sampled tissues were also subjected to histopathological analysis. Tissues fixed in 10% neutral buffered formalin (Fujifilm Wako Pure Chemical Corporation) were processed according to routine methods, then embedded in paraffin wax. Sections were stained with haematoxylin and eosin for histopathological examination. Immunohistochemical staining was also performed using antigen retrieval solution, 0.05% citraconic anhydride, pH 7.4 (Immunosaver; Nissin EM, Tokyo, Japan), mouse anti-influenza A virus matrix protein monoclonal antibody (clone GA2B; Serotec Ltd., Oxford, UK), and the Simple Stain MAX-PO (M) kit (Nichirei Bioscience Inc., Tokyo, Japan), in accordance with the manufacturers’ instructions.

## Supplementary Information

**Additional file 1. **Profiles and virological/serological assessments of the captured ducks.

**Additional file 2. **Experimental designs.

## Data Availability

The datasets supporting the conclusions of this article are included within the article and its additional files.

## Abbreviations

cELISA: Competitive enzyme-linked immunosorbent assay
dpi: Days post-inoculation
EID_50_: 50% Egg infectious dose
HA: Hemagglutinin
HI: Hemagglutination inhibition
HPAI: High pathogenicity avian influenza
HPAIV: High pathogenicity avian influenza virus
IUCN: International Union for Conservation of Nature and Natural Resources
