# Supplementary material for: Susceptibility of common family Anatidae bird species to clade 2.3.4.4e H5N6 high pathogenicity avian influenza virus: an experimental infection study
Source: BMC Vet Res. 2022 Apr 2;18:127. doi: 10.1186/s12917-022-03222-7 (PMC8976319; doi:10.1186/s12917-022-03222-7)
Supplement: Supplementary file 1 — Additional file 1. Profiles and virological/serological assessments of the captured ducks. [file 12917_2022_3222_MOESM1_ESM.pptx]

## Slide 1
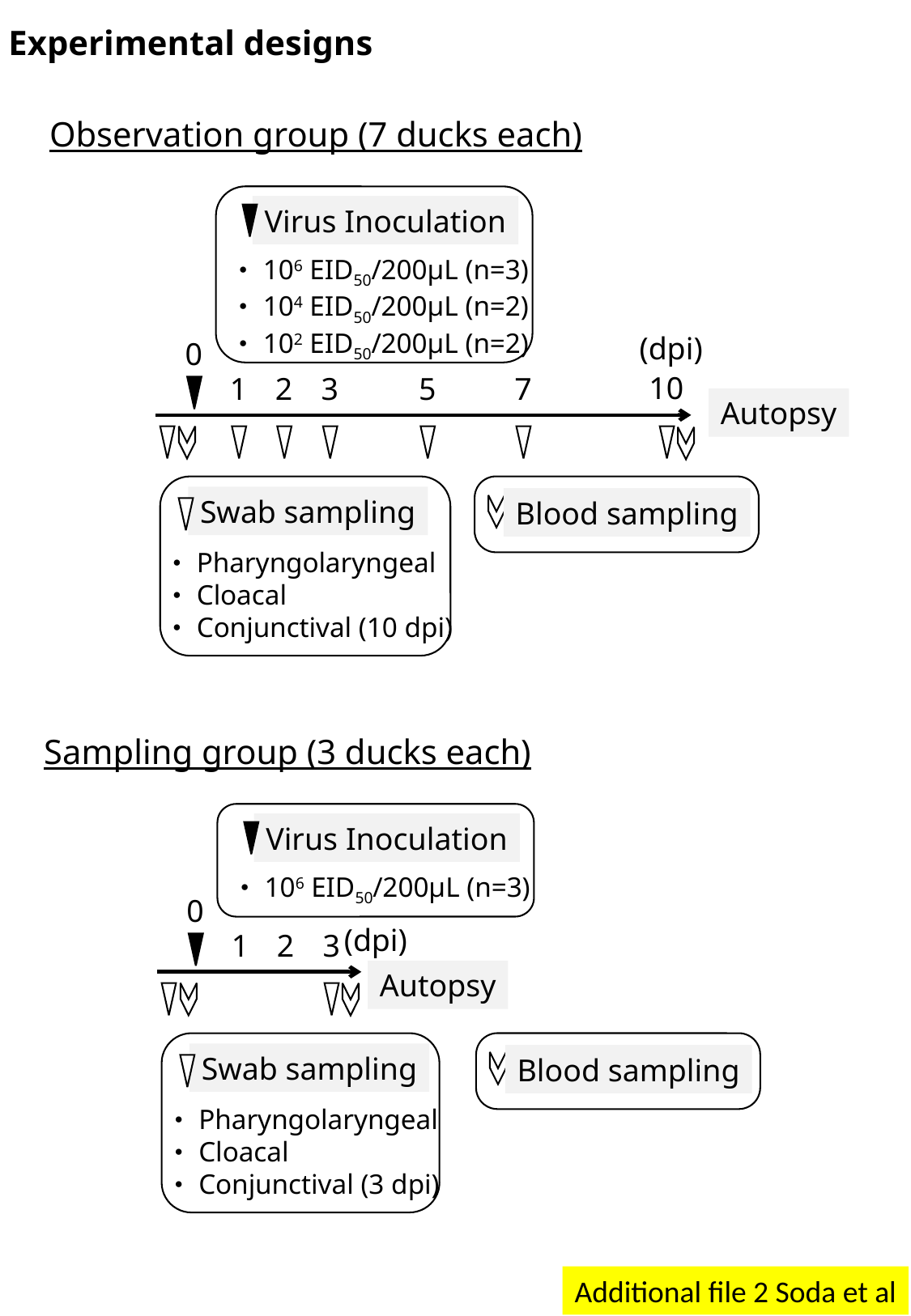

Experimental designs
Observation group (7 ducks each)
Virus Inoculation
・106 EID50/200μL (n=3)
・104 EID50/200μL (n=2)
・102 EID50/200μL (n=2)
(dpi)
0
10
1
2
3
5
7
Autopsy
Swab sampling
Blood sampling
・Pharyngolaryngeal
・Cloacal
・Conjunctival (10 dpi)
Sampling group (3 ducks each)
Virus Inoculation
・106 EID50/200μL (n=3)
0
(dpi)
1
2
3
Autopsy
Swab sampling
Blood sampling
・Pharyngolaryngeal
・Cloacal
・Conjunctival (3 dpi)
Additional file 2 Soda et al
